# Thoracotomy versus video-assisted thoracoscopic resection of lung cancer: A protocol for a systematic review and meta-analysis

**DOI:** 10.1097/MD.0000000000014646

**Published:** 2019-03-08

**Authors:** Tianci Chai, Yuhan Lin, Mingqiang Kang, Jiangbo Lin

**Affiliations:** a Department of Thoracic Surgery, Fujian Medical University Union Hospital; b The Graduate School of Fujian Medical University; c School of Stomatology, Fujian Medical University, Fuzhou, China.

**Keywords:** cancer resection, lung, thoracotomy, video-assisted thoracoscopic surgery

## Abstract

**Background:**

Video-assisted thoracoscopic surgery (VATS) is a kind of minimally invasive surgery with the advantages of small surgical incision, less surgical bleeding, and fewer hospitalization days. However, traditional thoracotomy has advantages in lymph node dissection and radical resection of tumors and the benefits of VATS compared with thoracotomy for lung cancer are controversial. This systematic review and meta-analysis will be conducted to evaluate the advantages and disadvantages of the 2 different surgical methods.

**Methods and Analysis:**

PubMed (Medline), Embase, Cochrane Central Register of Controlled Trials, and Google Scholar will be searched for relevant randomized controlled trials (RCTs), quasi-RCTs, and Hi-Q (high quality) prospective cohort trials published or unpublished in any language before March 1, 2019. Subgroup analysis will be performed in type of operation, tumor pathological stage, and ethnicity.

**Results:**

The results of this study will be published in a peer-reviewed journal.

**Conclusion:**

As far as we know, this study will be the first time to compare and meta-analyze the efficacy of thoracoscopic lung cancer resection and thoracotomy. This study will provide high-quality and reliable evidence for clinicians’ decision-making by comparing published or completed but unpublished trials data. Because of the characteristics of disease and intervention methods, large sample size and RCTs may be insufficient. We will carefully consider the inclusion of small sample RCTs, but this may lead to high heterogeneity and affect the reliability of research results.

**PROSPERO registration number:**

CRD42018118427

## 1 Introduction

Lung cancer is one of the most common malignant tumors and the main cause of cancer-related death.^[1,2]^ Surgery can provide a potential cure opportunity for patients with lung cancer. In the past decade, video-assisted thoracoscopic surgery (VATS) has made great progress and become the most frequently used surgical procedure for lung cancer patients worldwide.^[3,4]^ VATS has the advantages of smaller incision, less postoperative pain, fewer complications and shorter hospitalization time compared with thoracotomy.^[5–12]^ However, it also has some disadvantages, such as less surgical space and incomplete lymph node dissection.^[13,14]^ In addition, repeated rotation of the lens and ultrasonic scalpel may lead to the fragmentation and spread of tumors as well as bronchial or vascular injury. On the contrary, thoracotomy can offer a reasonable access to all areas of the thorax for surgeons and allow them to accomplish anatomic resections and wedge resections with direct observation of lesion area.^[13,14]^

The benefits of VATS for lung cancer patients are still controversial. This study will compare the efficacy and outcome of the 2 different surgical methods to determine which one is more likely to benefit patients with lung cancer, and provide a basis for clinicians to develop optimal treatment strategies for patients with lung cancer.

## 2 Objective

We will conduct a systematic review and meta-analysis to estimate the efficacy of VATS versus thoracotomy for patients with lung cancer.

## 3 Methods

This protocol is performed adhere to the Preferred Reporting Items for Systematic Review and Meta-Analysis Protocols (PRISMA-P) statement.^[15]^ The results of this systematic review and meta-analysis will be published with reference to the Preferred Reporting Items for Systematic Reviews and Meta-Analyse (PRISMA) guidelines.^[16]^ This protocol has been registered in the PROSPERO network (registration number: CRD42018118427).

### 3.1 Eligibility criteria

#### 3.1.1 Types of studies

Randomized controlled trials (RCTs), quasi-RCTs, and Hi-Q (high quality) prospective cohort trials published or unpublished will be included that should be completed and compare thoracotomy versus video-assisted thoracoscopic resection of lung cancer.

#### 3.1.2 Types of participants

The participants will be patients diagnosed with resectable lung cancer pathologically confirmed, who were treated surgically and there will be no restrictions on sex, ethnicity, economic status, and education.

#### 3.1.3 Types of interventions

All types of thoracotomy compared with VATS resection of lung cancer for patients who were diagnosed with resectable lung cancer.

#### 3.1.4 Types of outcome measures

##### 3.1.4.1 Primary outcomes

The primary outcome will be overall survival of patients with resectable lung cancer after surgery.

##### 3.1.4.2 Secondary outcomes

We will evaluate the 5-year survival, recurrence-free survival, median survival, quality of life, and complication rate of patients with resectable lung cancer after surgery.

### 3.2 Information sources

Two reviewers (TCC, YHL) will search PubMed, Cancerlit, Embase, Cochrane Central Register of Controlled Trials, and Google Scholar for relevant trials published before February 20, 2019, without any language restrictions.

### 3.3 Search strategy

The subject terms and keywords corresponding to Medical Subject Heading (MeSH) terms will be used to search for eligible trials in the databases as mentioned above with no language restrictions. Search strategies in PubMed are summarized in Table 1.

**Table 1:**
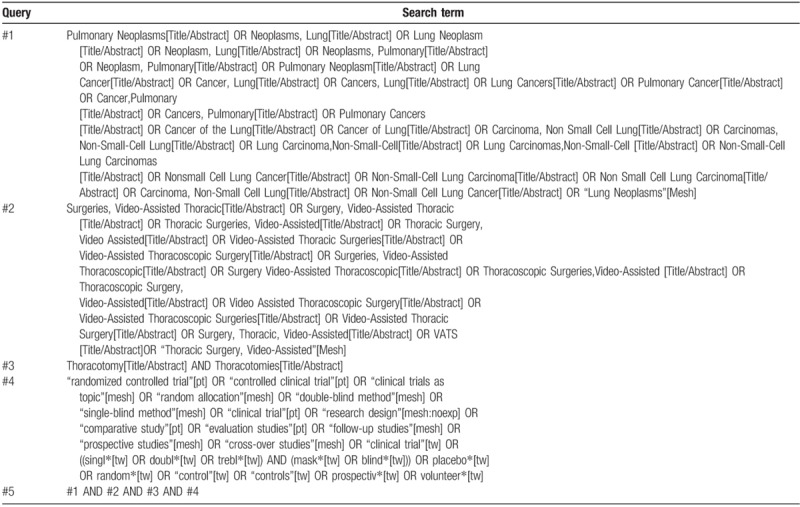
PubMed search strategies.

### 3.4 Data collection and analysis

We will adopt the methods described in the Cochrane Handbook for Systematic Reviews of Interventions to pool the evidence.^[17]^

#### 3.4.1 Study selection

Two authors (TCC, YHL) will screen independently each title and abstract of all the papers searched and the trials do not meet the inclusion criteria described in this protocol will be excluded. Full text of all the possible eligible trials will be screened independently and in duplicate by the 2 authors. Trials that are irrelevant or do not meet the inclusion criteria will be excluded. Trials that meet the inclusion criteria and excluded studies with the reasons for their exclusion will be documented by 2 authors (TCC, YHL). If there is a disagreement between the 2 authors, we will resolve the disagreement by discussing with the third author (JBL). If necessary, we will consult the fourth author (MQK) to resolve the disagreement. Selection process will be shown in PRISMA flow chart in details.

#### 3.4.2 Data extraction and management

We will extract the following data from the trials included.

(1) Study characteristics: author, publication date, country, study design, randomization, periods of data collection, follow-up duration, withdrawals, and overall duration of study.
(2) Population characteristics: age, sex, pathology diagnosis, tumor stage, pathologic tumor size, performance status, ethnicity, history of smoking, and inclusion criteria.
(3) Interventions: type of operation, number of lymph nodes retrieved, extent of resection, duration of operation, bleeding, postoperative adjuvant therapy.
(4) Outcomes: overall survival, 5-year survival, recurrence-free survival, median survival, length of stay, length of ICU stay, quality of life, complications, adverse events.

We will use the pre-designed table to record the data extracted from the included trials. If relevant data of the trials are lost or unclear, we will consult the author via email before determining whether the study is included.

### 3.5 Assessment of risk of bias

The Cochrane Handbook for Systematic Reviews of Interventions will be used to assess the risk of bias of each trial included. The 2 authors (TCC, YHL) will evaluate the risk of bias based on the following domains: random sequence generation (selection bias), allocation concealment (selection bias), blinding of participants and personnel (performance bias), blinding of outcome assessment (detection bias), incomplete outcome data (attrition bias), selective outcome reporting (reporting bias), and other bias.^[18]^ The risk of bias in each domain will be assessed as high, low, or uncertain, and the results of the evaluation will be shown on the risk of bias graph.

### 3.6 Data analysis

We will use Review Manager 5.3 software to synthesize the data extracted. If the data extracted from the included studies are evaluated as highly homogeneous, we will conduct a meta-analysis on them for the purpose of obtaining a clinically meaningful result. In order to carry out a standard meta-analysis, we will use the Chi^2^ and *I*^2^ statistic test to evaluate statistical heterogeneity among the studies. If there is high heterogeneity (*P* < .1 or *I*^2^ statistic > 50%), we will use the random effect model to analyze the extracted data. Otherwise, we will adopt fixed-effect model to analyze the data. We will adopt the Mantel–Haenszel method to pool the binary data and the results will be reported in the form of relative risk (RR) within the 95% confidence interval (95% CI). Inverse variance analysis method will be used to pool the continuous data and the results will be reported in the form of standardized mean difference (SMD) within 95% CI.

#### 3.6.1 Subgroup analysis

If there is substantial heterogeneity and the available data are sufficient, we will perform subgroup analysis for searching potential origins of heterogeneity. If the extracted data are enough, we will conduct subgroup analysis of the type of operation, tumor stage, age, and postoperative adjuvant treatment.

#### 3.6.2 Sensitivity analysis

We will conduct sensitivity analysis to evaluate the robustness and the reliability of aggregation results by eliminating trials with high bias risk.

### 3.7 Publication bias

Funnel charts and Egger test will be adopted to assess publication bias if there are no less than 10 eligible trials. If publication bias is suspected in a trial, we will consult the corresponding author via email to determine whether there is publication bias. If publication bias exists, we will use the methods of fill and trim to analyze publication bias.^[19]^

### 3.8 Evidence evaluation

We will classify the quality of all evidence into 4 levels (high, medium, low, and very low) in accordance with the criteria of GRADE (study limitations, imprecision, publication bias, indirectness bias, and effect consistency).^[20]^

## 4 Discussion

Recent research shows that the incidence of lung cancer worldwide has been increasing year by year and has become the highest incidence of malignant tumors,^[1]^ and is also the main cause of cancer-related deaths. Surgical resection of lung cancer can effectively improve the overall survival rate of patients and may provide a cure opportunity for lung cancer patients.

In recent decade, VATS technology has been rapidly developed and widely used in the treatment of lung cancer. Compared with traditional thoracotomy, VATS has less postoperative pain, fewer complications, shorter hospitalization days, and is more conducive to postoperative adjuvant treatment.^[21–25]^ However, surgeons cannot directly observe the lesions that are not detected by imaging examination are easily overlooked by surgeons. Because of the limited operating space of VATS, it has defects in lymph node dissection and radical resection of large tumors. On the contrary, thoracotomy allows the operator to have a direct observation and contact with the surgical field, which facilitates lymph node dissection and complete resection of large or advanced tumors.^[13,14]^

Both of these surgical methods are the main treatment for lung cancer resection, but which one is more beneficial to lung cancer patients is still controversial. As some high-quality related studies have been published or are in progress, we will conduct a systematic review and meta-analysis of these high-quality clinical studies to evaluate which method can benefit lung cancer patients more. The results of this study will provide a basis for the formulation of clinical treatment strategies for patients with lung cancer.

## Author contributions

Jiangbo Lin and Mingqiang Kang is the guarantor of the article. Tianci Chai and Yuhan Lin conceived and designed the study. Tianci Chai and Yuhan Lin drafted this protocol. Tianci Chai, Jiangbo Lin, and Yuhan Lin will perform the search, screening, and extraction. Jiangbo Lin and Mingqiang Kang have strictly reviewed this protocol and approved of publication. Tianci Chai and Yuhan Lin contributed equally to this work.

**Conceptualization:** Tianci Chai, Mingqiang Kang.

**Data curation:** Tianci Chai, Yuhan Lin.

**Formal analysis:** Tianci Chai.

**Funding acquisition:** Jiangbo Lin.

**Investigation:** Tianci Chai.

**Methodology:** Tianci Chai.

**Project administration:** Jiangbo Lin.

**Software:** Tianci Chai, Yuhan Lin.

**Supervision:** Mingqiang Kang, Jiangbo Lin.

**Validation:** Tianci Chai.

**Visualization:** Tianci Chai.

**Writing – original draft:** Tianci Chai, Yuhan Lin.

**Writing – review & editing:** Mingqiang Kang, Jiangbo Lin.
